# The Importance of Physiologically Relevant Cell Lines for Studying Virus–Host Interactions

**DOI:** 10.3390/v8110297

**Published:** 2016-11-01

**Authors:** David Hare, Susan Collins, Breanne Cuddington, Karen Mossman

**Affiliations:** 1 Pathology and Molecular Medicine, McMaster University, 1280 Main Str. West, Hamilton, ON L8S 4L8, Canada; haredn@mcmaster.ca (D.H.); susan_collins@hotmail.com (S.C.); cuddinbp@mcmaster.ca (B.C.); 2 Biochemistry and Biomedical Sciences, McMaster University, 1280 Main Str. West, Hamilton, ON L8S 4L8, Canada

**Keywords:** primary cell, immortalized cell, interferon, interferon regulatory factor 3, interferon-stimulated genes, signal transduction, antiviral defense

## Abstract

Viruses interact intimately with the host cell at nearly every stage of replication, and the cell model that is chosen to study virus infection is critically important. Although primary cells reflect the phenotype of healthy cells in vivo better than cell lines, their limited lifespan makes experimental manipulation challenging. However, many tumor-derived and artificially immortalized cell lines have defects in induction of interferon-stimulated genes and other antiviral defenses. These defects can affect virus replication, especially when cells are infected at lower, more physiologically relevant, multiplicities of infection. Understanding the selective pressures and mechanisms underlying the loss of innate signaling pathways is helpful to choose immortalized cell lines without impaired antiviral defense. We describe the trials and tribulations we encountered while searching for an immortalized cell line with intact innate signaling, and how directed immortalization of primary cells avoids many of the pitfalls of spontaneous immortalization.

## 1. Introduction

As an obligate intracellular parasite, a virus’s success depends on strategies evolved to exploit host cells and the suitability of these strategies to overcome cellular antiviral defenses. Intrinsic antiviral defenses include passive features that protect the host from infection. For example, the cell membrane separates the intracellular space from the environment and creates a barrier to invading pathogens. In addition to intrinsic barriers to infection, cells have innate immune defenses activated by pathogen recognition. Viruses must overcome both intrinsic and innate barriers to infection, interacting with the cell to avoid or impair the cell’s defenses.

The major component of innate antiviral defense is the type 1 interferon (IFN-I) system. The ability of a virus to prevent IFN-I production is critical for replication and is an important determinant of a virus’s species tropism [1]. The IFN-I family comprises a group of innate cytokines that bind the IFN-I receptor (IFNAR) and have antiviral and immunomodulatory effects. IFNs are usually released following recognition of virus-associated molecular patterns, such as viral nucleic acid, by pattern recognition receptors (PRR). The best characterized PRRs include toll-like receptors (TLRs), which recognize nucleic acids or proteins with viral signatures in the endosome, RIG-I-like receptors (RLR), which recognize cytosolic double-stranded and 3′-triphosphorylated RNA molecules, and DNA receptors which recognize mis-localized or foreign DNA [2]. In addition to pathogen molecules, certain cell stress signals can also activate innate immunity [3]. Binding of virus-associated molecules by PRRs or recognition of cellular stress signals IFN-I induction through the interferon regulatory factor (IRF) family of transcription factors. Upon activation, IRF3 forms a homodimer and translocates to the nucleus to induce the expression of IFN-β. Activated IRF3-IRF7 heterodimers translocate to the nucleus and induce the expression of several IFN-α subtypes. Binding of IFN-α/β to IFNAR activates both signal transducer and activator of transcription 1 (STAT1) and STAT2 which, together with IRF9, form the IFN-stimulated gene factor (ISGF3) complex. This complex enters the nucleus and binds the IFN-stimulated response element (ISRE) to up-regulate the expression of IFN-stimulated genes (ISGs). There are several reviews of this pathway that go into more detail [4,5,6].

IRF3 plays a critical role in IFN-I induction but is also critical for parallel IFN-independent responses. Canonical IRF3 activation involves phosphorylation of serine 386 and serine 396 clusters by TANK Binding Kinase 1 (TBK1), followed by IRF3 dimerization, nuclear translocation and DNA binding [7]. However, IRF3 activation markers are often inconsistent in response to different stimuli suggesting alternate modes of IRF3 activation exist [8]. Furthermore, different modes of IRF3 activation allow cells to respond accordingly to the severity of infection [9]. For example, infection with low particle numbers of non-replicating enveloped virus induces a subset of ISGs in an IRF3-dependent IFN-independent manner [10,11]. This response is hypothesized to act as a first line of defense during initial stages of virus infection before subsequent rounds of viral replication. Since the IFN-independent response is particularly tuned to low-level infection, only cells with intact antiviral defense pathways are able to respond properly. While tumor-derived cell lines may retain the ability to respond to IFN-I and potent IFN-I inducers, like replicating Sendai virus (SeV) and the synthetic double stranded RNA (dsRNA) analog polyinosinic-polycytidylic acid (poly I:C), most do not mount an IFN-independent response to low-level infection.

IFN-β activity is nearly undetectable in unstimulated cells because aberrant ISG expression can be harmful and is associated with autoimmune disease. However, low-level basal IFN-β signaling plays an important role in priming cells to rapidly and robustly respond to infection and maintain homeostasis [12,13]. For example, while IRF3 is constitutively expressed and quite stable, the more labile IRF7 requires a basal IFN-β signaling loop for its expression and is maintained at low to undetectable levels [14,15]. Basal IFN-β signaling regulates a number of ISGs involved in antiviral defense, virus recognition and induction of IFN-α. This basal activity allows signaling components upregulated by IFN-β to amplify the response to virus infection. However, cells lacking such signaling also lack basal expression of important genes that regulate the induction of IFN-β.

## 2. The Challenges of Primary Cells

An ideal in vitro system should be tractable and representative of cells in vivo. Primary cells better resemble the healthy cells of their tissue of origin because they have not accumulated mutations that arise during tumorigenesis or continual passage in tissue culture. When studying viruses, the way they interact with their host is critical, and differences in the cellular model may lead researchers to false conclusions. While primary cells are more representative of the tissue they were isolated from, they are not a particularly tractable system. Stable expression of exogenous genes in primary cells is virtually impossible because they are limited to a certain number of population doublings and are often approaching senescence by the time selection is complete. In addition to the hallmarks of senescence, including cell cycle arrest, cell enlargement and resistance to apoptosis, cells at later passage number and approaching senescence express higher basal levels of IFN-β, and consequently mount a stronger IFN-I response to virus infection [16,17]. The phenotypic changes that occur in primary cells at later passage can impact virus–host interactions and consequently passage-matched cells are required for experiments. Another difficulty is that primary cells are often resistant to transfection with plasmid DNA, making transient expression of exogenous genes difficult. The low transfection efficiency of primary cells is often attributed to intrinsic and innate pathways activated by foreign DNA. Interferon gamma inducible protein 16 (IFI16), promyelocytic leukaemia (PML) bodies, and other intrinsic factors recognize foreign DNA in the nucleus and epigenetically repress gene expression [18,19]. In addition to intrinsic defenses against exogenous gene expression, transfection of DNA or dsRNA is able to induce innate responses through the IFN-I pathway [20,21,22]. This pitfall can be mitigated by inclusion of appropriate controls to rule out the effects of transfection alone. However, the background antiviral response under these conditions can potentially confound interpretation of results, particularly at low levels of stimulation.

The difficulties associated with manipulating primary cells make the use of other models a necessity. Transformed cells are generally much easier to manipulate in culture because they have higher transfection efficiency, faster growth rate, and do not senesce. These qualities allow for the creation of cell lines suited to answering difficult research questions and are critical for a deeper understanding of virus–host interactions. However, transformed cells often do not resemble the original cell type. Cells accumulate mutations during tumorigenesis and continual culture that over time affect the phenotype and how cells respond to virus infection. Furthermore, innate signaling pathways may overlap with senescence pathways, making dysregulation of these pathways a natural consequence of spontaneous immortalization. Consequently, when the IFN-β signaling loop is dysregulated in immortalized cell lines, these cells have a broadly impaired antiviral response.

## 3. Cellular Immortalization

There are multiple paths to cellular immortalization. First, cells must avoid senescence—a state where cells undergo replicative arrest—remain metabolically active and survive in culture for long periods of time. Senescence is controlled by molecular clocks within the cell which measure age, and perhaps the most important of these clocks are the telomeres [23,24,25]. These regions of heterochromatin capping each chromosome become shorter each time the genome is replicated. Telomeres can be maintained in stem cells and other dividing cells by expression of the enzyme telomerase, which catalyzes template-dependent addition of telomeric repeats [26]. Telomerase contains telomerase RNA template component (TERC) and telomerase reverse transcriptase (TERT) [27]. In the absence of telomerase, telomeres become critically short and the tumor suppressor p53 and retinoblastoma protein (pRb) halt the cell in G phase of cell cycle [28]. While replicative senescence in human cells appears to be dependent on telomere shortening, there may be additional aging clocks and some differences in replicative senescence exist in non-human cells [29]. Tumorigenesis and spontaneous immortalization usually begin with inactivation of one or both of p53 or pRb. Similarly, viral oncogenes often target one of these two pathways to drive the cell cycle forward [28]. Although loss of p53 or pRb is sufficient to bypass senescence, it doesn’t prevent the shortening of telomeres, and at a certain point cells enter a stage called “crisis” where the cell population declines because of widespread apoptosis [30]. All of the clones which emerge from crisis with a truly immortal phenotype have gained the ability to stabilize telomere length, either by expression of functional telomerase or by a recombination-driven process called alternative lengthening of telomeres.

## 4. Effect of Immortalization on Antiviral Signaling

To immortalize cells in a way that preserves innate signaling pathways, it is important to understand how innate signaling pathways are dysregulated during immortalization and transformation. There is a great deal of overlap between cellular pathways involved in antiviral defense and tumor suppression. Both pathways can trigger anti-proliferative, pro-apoptotic and pro-inflammatory responses. It has been hypothesized that tumor suppressors and antiviral defense share a common evolutionary history and that many tumor suppressors first evolved as a defense against DNA viruses [16,31,32]. In support of this hypothesis, cells lacking antiviral genes are more easily immortalized in culture [33]. Additionally, cells lacking tumor suppressors are more susceptible to virus infection [34,35]. Antiviral genes may be silenced by the loss of certain tumor suppressors or selected against during tumorigenesis due to their anti-proliferative nature. Immortalization is a complex and often stochastic process, but certain defects are commonly linked with the loss of antiviral responsiveness.

p53 is an important tumor suppressor lost in most spontaneously immortalized and transformed cells. Nuclear p53 is activated by DNA damage and acts as a transcription factor for various anti-proliferative and apoptotic genes [36]. Additionally, cytosolic p53 can induce transcription-independent apoptosis by interacting at the mitochondria with B-cell lymphoma 2 (Bcl2) [37]. Not only is p53 an important tumor suppressor, but it also plays a role in antiviral immunity by halting growth or triggering apoptosis during virus infection [38]. A number of viruses, including simian virus 40 (SV40), adenovirus, human herpesvirus-8 (HHV-8) and papillomavirus, encode p53 antagonists, highlighting the importance of p53 during antiviral defense [32]. In fact, p53 was initially discovered as a 53 kDa protein co-precipitated with the SV40 large T antigen (LTA) [39]. In addition to halting cell cycle and initiating apoptosis, p53 also regulates IFN-I signaling. When immortalized p53^−/−^ fibroblasts from Li-Fraumeni syndrome (LFS) patients were compared to non-immortalized controls, many IFN-I stimulated genes were down regulated [40,41]. Although most ISGs do not have p53 responsive elements in their promoters, IRF9 is an important innate signaling protein controlled in part by p53 [42]. As IRF9 is essential for signaling downstream of IFN-I, down-regulation of IRF9 in p53^−/−^ cells would interfere with basal IFN-β signaling. Thus, the loss of p53 indirectly impairs the ability of cells to induce and respond to IFN-I during a viral infection.

pRb is another important tumor suppressor that plays a role in antiviral signaling. In addition to regulating proteins involved in cell cycle progression, pRb regulates a number of immune signaling proteins involved in the induction and response to cytokines [43]. pRb knockout cells have a number of defects including reduced TLR3 expression, impaired NF-kB activation, and increased permissivity to vesicular stomatitis virus (VSV) [44,45]. Furthermore, the pRb-binding domains of certain oncogenic viral proteins are important for antagonizing innate signaling, suggesting pRb regulates the innate signaling pathways targeted by these viruses. Polyoma virus with a mutant T antigen protein unable to bind pRb was also unable to block Jak-STAT1 signaling downstream of IFN-I [46]. The pRb-binding domain of papillomavirus E7 is required for its ability to repress IRF1 downstream of IFN-γ signaling [47]. Finally, the pRb-binding domain of adenovirus early region 1A (E1A) is required for it to suppress nuclear factor kappa B (NF-kB) activation downstream of tumor necrosis factor (TNF)-α [48]. Thus, pRb plays a number of distinct roles in innate signaling which explains why pRb-deficient cells are more permissive to virus infection.

IRF1 and Ras play an interconnected role in tumorigenesis with implications for innate signaling. IRF1 is a transcription factor first identified to regulate IFN-β expression and a subset of ISGs, but is lost in a variety of cancers [49]. Overexpression of IRF2, which antagonizes IRF1, promotes transformation of NIH 3T3 cells, suggesting that IRF1 is a tumor suppressor [50,51]. The Ras family of GTPases are involved in a variety of pathways involving cell proliferation and morphology and activating Ras mutations are observed in the majority of cancers [52]. Additionally Ras transformed cells have impaired IFN-α induction and enhanced permissivity to oncolytic viruses [53]. When murine embryonic fibroblasts (MEFs) were Ras-transformed in the presence of IRF1, it appeared to trigger p53-dependent growth-arrest and apoptosis; consequently, IRF1^−/−^ MEFs were more easily transformed [54,55]. Furthermore, it has been observed that Ras-transformed cells have down-regulated IRF1, which explains the reduced antiviral responsiveness of these cells [56,57]. Thus, IRF1 acts as a tumor suppressor during Ras-transformation and is selected against during tumorigenesis. The loss of IRF1 additionally leads to the downregulation of a number of antiviral ISGs involved in innate signaling. Although less is known about IRF5, it is also lost in certain cancer types, and exogenous IRF5 expression is sufficient to sensitize cells to DNA damage and inhibit tumor growth [58,59].

Although IFNAR1 is generally not considered a tumor suppressor, IFNAR1-deficient MEFs spontaneously immortalize in culture at a higher rate than normal MEFs, suggesting an important role of IFN-I signaling in tumor suppression [33]. Exogenous IFN-I is sufficient to suppress transformation and can induce senescence by upregulation of cyclin-dependent kinase inhibitors p15, p21, and p27 [38,60,61]. Furthermore, IFN-I signaling increases in MEFs as they approach senescence, with late passage MEFs producing higher basal levels of IFN-β than early passage MEFs [17]. Due to higher basal IFN-β signaling, late passage MEFs have higher levels of IFN-β induction in response to virus infection [17]. One explanation for the increase in basal IFN-β as cells age may be the response to DNA double-stranded breaks (DSB). When telomeres shorten past a certain point, the genome ends are recognized by the DNA DSB repair machinery and trigger senescence [62]. DNA DSBs induced by genotoxic agents or expression of a telomere-specific nuclease are sufficient to induce IFN-I [63,64]. Further, knockout of ataxia telangiectasia mutated (ATM), an important DNA DSB repair enzyme, primes the IFN-I response through a stimulator of interferon genes (STING)-dependent pathway [65]. These studies suggest that DNA DSBs that result from telomere shortening or DNA damaging agents may be sensed by DNA sensors that in turn activate STING and IFN-I production. The two best-characterized DNA sensors upstream of IFN-I induction are cyclic GMP-AMP synthase (cGAS), which non-specifically recognizes DNA in the cytosol, and IFN-γ inducible protein 16 (IFI16), which is believed to recognize naked DNA in the nucleus [66,67]. It is not clear whether either of these sensors is involved in recognition of DNA DSBs or shortened telomeres.

In addition to the in vitro role of IFN-I in senescence it may also play a role in vivo. Progeria is a disease of accelerated aging caused by defects in telomerase or other maintenance enzymes, and stem cells from progeria patients rapidly approach senescence. However, replication of stem cells from progeria patients can be rescued from senescence by treatment with anti-IFN-β antibodies [63]. While mice lacking the telomerase component TERC show signs of accelerated aging, this phenotype is reversed in double knockouts additionally lacking the IFN-I receptor component IFNAR1 [63].

## 5. Developing a Cellular Model to Study Virus–Host Interactions: Trials and Tribulations

To study the IFN-independent antiviral response to low-level virus infection, a sufficiently sensitive cell type must be used because of the subtle nature of the stimuli and the sensitivity of the response to cell stress. Unfortunately, defects in innate antiviral signaling present in cancer cell lines impact both IFN-I signaling and the IFN-independent response. To identify an immortalized cell type in which to study IFN-independent antiviral responses, we screened a number of candidates from the NCI-60 panel of human tumor cell lines for their ability to mount an antiviral response following infection with ultraviolet (UV)-inactivated RNA or DNA viruses. There was a high degree of variability between cell lines, with some responding to both, one, or none of the UV-inactivated viruses (unpublished data). The existence of cell lines responsive to multiple UV-inactivated viruses shows that tumorigenesis does not necessarily result in loss of IFN-independent signaling. Furthermore, the varied antiviral responses of different cell lines suggest that innate signaling is lost through a variety of different mutations selected for during tumorigenesis.

In addition to screening cancer cell lines, we previously immortalized IRF3^+/+^ and IRF3^−/−^ primary MEFs derived from C57Bl/6 mice. We used the 3T3 protocol, which is an established technique for immortalization involving continual culture [68]. During continuous culture of MEFs, most cells senesce and the population goes through a period of little to no growth, but eventually some cells accumulate sufficient mutations to escape senescence and rapidly expand. Interestingly, IRF3^−/−^ MEFs spontaneously immortalized more readily using the 3T3 protocol, suggesting that IRF3 may play a suppressive role in immortalization (unpublished data). To test whether 3T3-immortalized MEFs mount an IFN-independent antiviral response we infected several immortalized MEF clones with UV-inactivated RNA and DNA viruses, and tested their ability to mount an antiviral response. All immortalized MEFs had defects in their antiviral response to virus particles (unpublished data). Similar to our own experiments testing the IFN-independent response, Wang et al. observed that 3T3 immortalized MEFs had impaired IFN-I activity following myxoma virus challenge, making them permissive to infection [69]. Interestingly, they did not observe impairment in IFN-I signaling, instead the immortalized MEFs had an impaired ability to produce IFN-β in response to myxoma virus infection.

The defects in the innate antiviral response of cancer cell lines and spontaneously immortalized MEFs highlight the care that must be taken during immortalization to preserve antiviral signaling. An alternative to spontaneous immortalization is exogenous expression of human telomerase reverse transcriptase (hTERT). hTERT is the limiting component of the telomerase enzyme and is deficient in most primary cells thereby limiting telomerase activity. However, exogenous expression of hTERT stabilizes telomere length, thus bypassing replicative senescence in a number of different cell types [70,71]. Exogenous hTERT was initially considered insufficient to immortalize certain cell types including keratinocytes and mammary epithelial cells, but senescence in these cells appears to be an artifact of tissue culture and can be rescued using feeder cells [72]. Fibroblasts were the original cell type immortalized using hTERT and hTERT-immortalized fibroblasts closely resemble primary cells, have intact p53/pRb signaling, and have normal responses to serum starvation, contact inhibition, or DNA damage [73,74]. Importantly, hTERT immortalized fibroblasts can undergo greater than 100 population doublings under the right conditions and exogenous hTERT expression does not impact IFN-I signaling and permissivity to herpex simplex virus (HSV) or VSV [75]. However, the effects on antiviral signaling of hTERT-immortalization in other cell types or following continual passaging have not been directly investigated.

Expression of viral-derived oncogenes like adenovirus E1A/E1B, papillomavirus E6/E7, or SV40 LTA is also used to immortalize primary cells. These oncogenes antagonize the tumor suppressors p53 or pRb, thereby preventing replicative senescence triggered by telomere shortening [28]. While viral oncogenes have been used to immortalize many commonly used cell lines, they also interfere with innate signaling. As previously discussed, the loss of p53 will negatively impact antiviral defense. Furthermore, viral oncogenes often target innate signaling pathways in addition to p53 and pRb. For example, papillomavirus E6 binds to IRF3 and prevents the induction of IFN-β [76], while adenovirus E1A protein is able to block downstream IFN-I signaling by binding and sequestering cAMP response element-binding (CREB)/p300 [77,78]. SV40 LTA-transformed cells have an up-regulated subset of ISGs but an impaired response to exogenous IFN-I [79]. This is because SV40 LTA expression activates a DNA damage response that upregulates ISGs through IRF1 activation [80,81]. An additional drawback of oncogene-transformed cell lines is that they enter crisis when their telomeres become critically short [30]. The clones that emerge from crisis harbor additional mutations, which might confound future studies.

In our hands, hTERT-immortalized human fibroblasts mount an antiviral response to UV-inactivated virus, exogenous IFN-α, or the dsRNA mimetic poly I:C with similar efficiency as primary human fibroblasts. The reason hTERT immortalization leaves innate antiviral signaling intact may be the lack of selection pressures that silence antiviral pathways. As with any cell type, continually cultured cells will gradually accumulate mutations, and higher passage hTERT immortalized cells will resemble the original primary cells less. However, directed immortalization by expression of hTERT avoids the bottleneck that occurs during spontaneous immortalization.

## 6. Conclusions

While in vitro studies will never be able to recapitulate the totality and complexity of the host antiviral response to infection, understanding specific virus–host interactions at the molecular level requires the use of model systems that best reflect the status of the host organism. Alterations in any number of pathways involved in cell homeostasis can affect antiviral defense because of the complex and interconnected nature of innate signaling. Therefore, primary cultures derived from healthy cells are perhaps the most convincing in vitro model of in vivo host defense. However, the limited lifespan of primary cells imposed by replicative senescence makes certain experimental approaches impossible. Additionally, powerful new clustered regularly interspaced short palindromic repeats (CRISPR)-based approaches to genome engineering necessitate the use of life-extended or immortalized cell lines. Immortalized primary cells can provide an accurate model for studying virus–host interactions if they are derived in such a way that preserves the integrity of innate antiviral signaling. Different cell types have been immortalized by exogenous hTERT expression, and hTERT-immortalized fibroblasts have been observed to have intact IFN-I and IFN-independent antiviral responses [75]. Thus, with sufficient care and characterization, relevant studies can be performed to elucidate fundamental aspects of virus–host interactions.
